# Nanoplastics in agriculture: an opinion on their effects on tomato crop

**DOI:** 10.3389/fpls.2026.1801857

**Published:** 2026-04-13

**Authors:** Laura Hernández-Sánchez, Vianii Cruz-López, Mario J. Romellón-Cerino, Rosario Herrera-Rivera, Raquel Murillo-Ortiz, Heriberto Cruz-Martínez

**Affiliations:** 1 Tecnológico Nacional de México, Instituto Tecnológico del Valle de Etla, Santiago Suchilquitongo, Oaxaca, Mexico; 2 Tecnológico Nacional de México, Instituto Tecnológico de Villahermosa, Villahermosa, Tabasco, Mexico; 3 Facultad de Ciencias Físico-Matemáticas, Universidad Autónoma de Nuevo León, San Nicolas de los Garza, Nuevo León, Mexico

**Keywords:** germination, nanoplastics, size and shape, uptake and transport pathways, yield and fruit quality

## 1 Introduction

**Figure 1:**
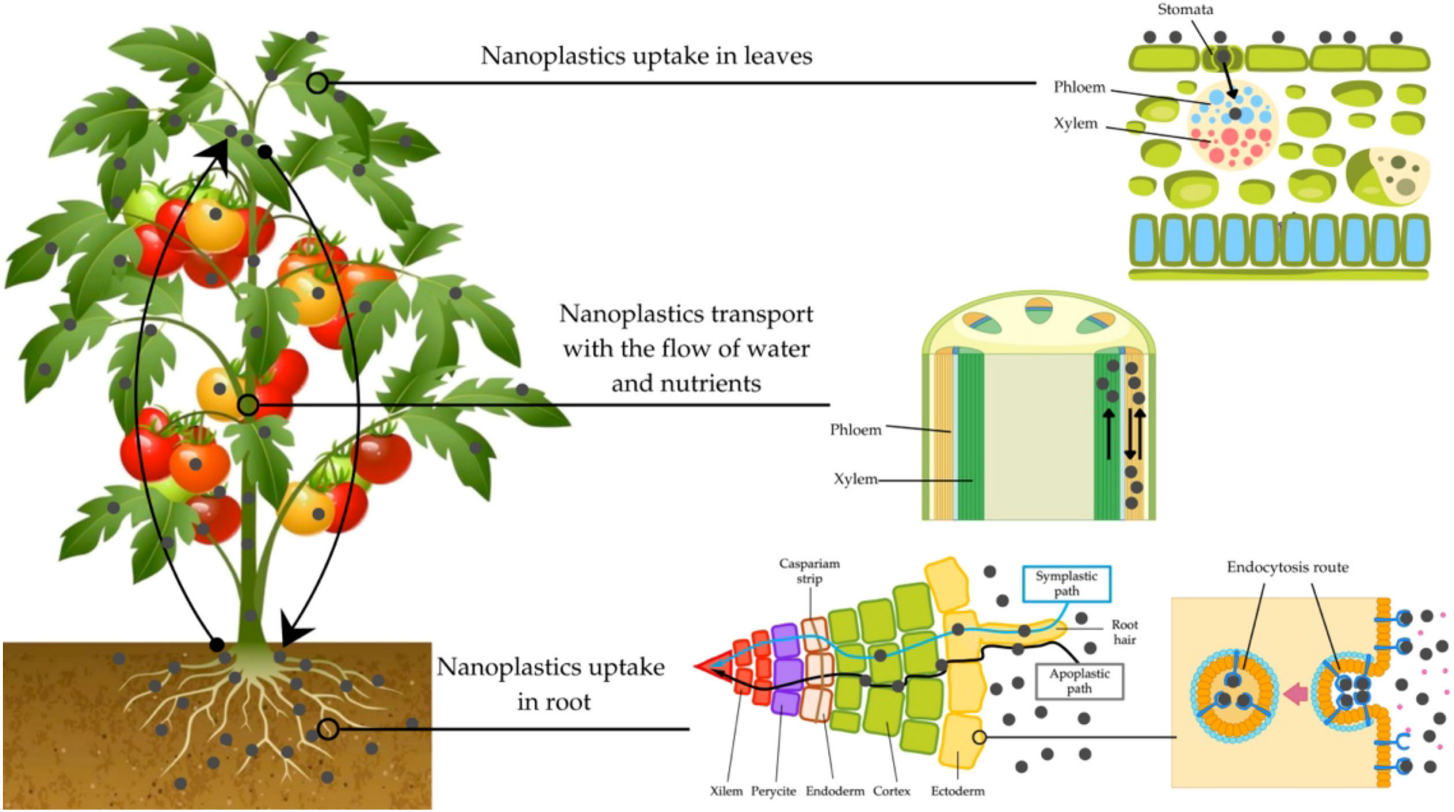
Possible uptake and transport pathways of nanoplastics in tomato plants.

The presence of nanoplastics (1–100 nm) in agricultural systems has emerged as a major concern due to their potential to contaminate soils and compromise plant growth and crop productivity in economically important crops (Ng et al., 2018; Chen et al., 2022; Santini et al., 2023; Hernández-Sánchez et al., 2025; Zytowski et al., 2025). These nanoparticles primarily originate from the bulk degradation of plastics, such as containers used for agricultural inputs and plastic mulching films applied in crop production (Li et al., 2022; Zytowski et al., 2025). The effects of nanoplastics on crops are particularly relevant because plants can absorb them through soil, water, or air. In soils, nanoplastics can modify key physical and chemical properties, including water retention, aeration, and nutrient availability, ultimately influencing crop growth and development (**Figure 1**) (Li et al., 2023). Reported investigations suggest that nanoplastics could penetrate crop cells and could exert pronounced effects on crop physiology (Ekner-Grzyb et al., 2022; Yu et al., 2024). The uptake and accumulation of nanoplastics in the edible parts of crops raise concerns about potential human exposure through food consumption (Zytowski et al., 2025). Nevertheless, studies in this field are still in their early stage.

Available studies investigating the effects of nanoplastics on the germination, growth, flowering, and yield of various crops suggest that nanoplastics may negatively influence different stages of plant development (Lian et al., 2020; Sun et al., 2022; Xie et al., 2025). To analyze the effects of nanoplastics on economically important crops, we selected tomato (*Solanum lycopersicum*), as it is the second most widely produced crop worldwide (Echeverría-Pérez et al., 2025). Furthermore, this crop provides essential components of the daily diet, including vitamins, carbohydrates, carotenoids, polyphenols, and minerals (Echeverría-Pérez et al., 2025). Therefore, we present our perspective on the current understanding of how nanoplastic characteristics and concentrations affect the different stages of tomato crop development and then discuss future directions for this topic.

## 2 Current understanding of the effects of nanoplastics on the tomato crop

### 2.1 Effects on the germination

Evidence regarding the effects of nanoplastics on tomato seed germination remains limited, with only a few studies available (Lakshmikanthan and Chandrasekaran, 2022a; Lakshmikanthan and Chandrasekaran, 2022b). These studies focused on exposing seeds to fluorescent (Lakshmikanthan and Chandrasekaran, 2022a) and non-fluorescent (Lakshmikanthan and Chandrasekaran, 2022b) spherical nanopolystyrene with sizes of 235.5 nm (Lakshmikanthan and Chandrasekaran, 2022a) and 149.5 nm (Lakshmikanthan and Chandrasekaran, 2022b), respectively. The results showed a pronounced reduction in germination performance, with treated seeds exhibiting germination rates of 63% for fluorescent nanopolystyrene (Lakshmikanthan and Chandrasekaran, 2022a) and 65.8% for non-fluorescent nanopolystyrene (Lakshmikanthan and Chandrasekaran, 2022b). In contrast, the control groups showed complete germination, suggesting that nanoplastics negatively impact seed germination (Lakshmikanthan and Chandrasekaran, 2022a; Lakshmikanthan and Chandrasekaran, 2022b). One possible explanation is that, due to their nanoscale size, these particles can readily penetrate seed coats and obstruct their pores, thereby reducing the uptake of oxygen and water, which are essential for germination (Bosker et al., 2019). However, these mechanisms focus on physical interactions with seed structures, and studies examining how nanoplastics affect the physiological and biochemical processes associated with seed germination are still needed.

### 2.2 Effects on the growth

Plastic nanoparticles can encounter plant roots, adhere to root surfaces, and enter root tissues through apoplastic and symplastic pathways or via endocytosis-mediated uptake. These particles can subsequently be translocated through the xylem vascular tissue, from roots to aerial plant organs, disrupting normal functions such as water and nutrient absorption, thereby affecting plant growth and development (**Figure 1**) (Hua et al., 2023). In this context, few studies have begun to assess the effects of different types of nanoplastics on tomato growth (Cao et al., 2024; You et al., 2026). Studies report that exposure to nanopolystyrene causes dose-dependent growth inhibition in tomato plants (Cao et al., 2024; You et al., 2026). For example, tomatoes grown in soil supplemented with nanopolystyrene (30 nm) with spherical shapes at concentrations of 150, 250, and 500 mg kg^-^¹ exhibited a significant, dose-dependent reduction in shoot and root fresh weight after 3 months of exposure compared to the control (Cao et al., 2024). Similarly, with different sizes (30 and 100 nm) at concentrations of 250 mg kg^-^¹, the same nanoplastic type shows that influence shoot and root fresh weight, with 30 nm nanoparticles having greater adverse effects than 100 nm nanoplastics, indicating that size plays a fundamental role in their effects on tomato crop growth (Cao et al., 2024). Consistent with these findings, it was reported that tomato plants exposed to nanopolystyrene (100 nm), applied at concentrations of 4, 8, 12, and 16 mg kg^-^¹, significantly reduced plant height, stem diameter, biomass, and leaf area, and that increasing concentrations of nanoplastics were associated with greater growth inhibition (You et al., 2026). Beyond dose-dependent effects, different varieties of the tomato crop also appear to modulate plant sensitivity to nanoplastics. In a recent study, six tomato varieties were exposed to nanopolystyrene (~50 nm) at 50 mg L^-^¹ for 2 weeks (Sun et al., 2025). Analyses of root, stem, and total dry biomass revealed growth inhibition in all varieties. However, Heinz 1706 showed the lowest reduction in total biomass (13%), while Moneymarker was the most sensitive, with a 32% reduction (Sun et al., 2025). Taken together, these findings highlight both the concentration-dependent nature of nanoplastic toxicity and the importance of varietal differences in determining plant responses to nanoplastics.

Exposure to nanoplastics can also have adverse effects on the biochemical and physiological processes of tomato plants. Specifically, reductions in total chlorophyll concentration of up to 22% occur in tomato plants treated with non-fluorescent (149.5 nm) and fluorescent (235.5 nm) nanopolystyrene compared to the control (Lakshmikanthan and Chandrasekaran, 2022a; Lakshmikanthan and Chandrasekaran, 2022b). Furthermore, nanopolystyrene exposure significantly reduced the net photosynthetic rate, transpiration rate, and stomatal conductance relative to the control (You et al., 2026). With respect to oxidative damage, exposure of tomato plants to nanopolystyrene across different particle sizes increases reactive oxygen species production and consequently antioxidant defense enzymes, such as catalase and superoxide dismutase, are activated (Lakshmikanthan and Chandrasekaran, 2022a; Lakshmikanthan and Chandrasekaran, 2022b; Cao et al., 2024; You et al., 2026). Notably, prolonged exposure times and high nanoplastic concentrations, such as nanopolystyrene (30 nm) at 500 mg kg^-^¹ for three months, resulted in elevated malondialdehyde and hydrogen peroxide levels, indicating cell membrane damage (Cao et al., 2024).

### 2.3 Effects on the yield and fruit quality

By altering water balance and nutrient uptake, nanoplastic accumulation can induce oxidative stress, ultimately affecting tomato fruit development and composition, and yield (Hernández-Sánchez et al., 2025). In this context, some investigations have evaluated the effects of nanoplastics on both fruit yield-related parameters and fruit quality attributes (Nazari et al., 2025; You et al., 2026). Regarding yield and fruit weight, recent research evaluated the effects of nanopolystyrene at two concentrations (4 and 16 mg kg^-^¹) and reported reductions in total yield and individual fruit weight, with more pronounced effects at higher nanoplastic concentrations (You et al., 2026). Similarly, when tomato plants were exposed to nanopolystyrene, particularly at higher concentrations (0.1 and 1%), a decrease in fruit fresh weight was obtained (Nazari et al., 2025).

In terms of fruit quality, tomato plants treated with nanopolyethylene (20–40 nm) for 50 days at nanoplastic concentrations of 0.01%, 0.1%, and 1% of soil weight showed significant alterations in fruit biochemical composition (Nazari et al., 2025). Specifically, there were reductions in soluble solids, total and reducing sugars, free amino acids, and lycopene content. In another study, the total soluble protein and vitamin C content decreased significantly at high concentrations (You et al., 2026). Notably, despite reduced fruit weight, exposure to higher nanopolystyrene concentrations increased fruit firmness and elevated levels of lycopene, flavonoids, and ascorbic acid—compounds closely associated with antioxidant capacity and nutritional quality (Nazari et al., 2025).

### 2.4 Effects on the genetics

Nanoplastics can penetrate plant tissues and interact with cellular and molecular components, potentially interfering with genomic integrity and modulating gene expression patterns in the tomato crops (Dehghanian et al., 2023; Hu et al., 2024; Hernández-Sánchez et al., 2025). At the transcriptional level, tomato plants exposed to nanopolyethylene (30 nm) showed pronounced changes in gene expression, with 790 genes differentially regulated relative to untreated controls, including 457 upregulated and 333 downregulated transcripts (Cao et al., 2024). In comparison, treatment with substantially larger micropolystyrene (50 μm) resulted in a markedly reduced transcriptional response, affecting only 439 genes (Cao et al., 2024). Beyond transcriptional effects, nanopolyethylene (20–40 nm) induced dose-dependent epigenetic modifications characterized by a non-linear methylation pattern. While an intermediate exposure level (0.1%) led to increased DNA methylation, higher concentrations (1%) caused widespread hypomethylation, potentially reflecting saturation or impairment of epigenetic maintenance systems under intense stress (Nazari et al., 2025). Consistent with these observations, nanoplastic-treated plants showed elevated expression of HDA3, a histone deacetylase involved in chromatin remodeling, and R2R3MYB, a transcription factor associated with defense signaling, whereas AP2a, a regulator of floral development and fruit maturation, was significantly repressed (Nazari et al., 2025). In another study, transcriptomic analysis of tomato seedling roots exposed to polystyrene nanoplastics (16 mg kg^-^¹) revealed significant alterations in gene expression, with 388 differentially expressed genes (247 upregulated and 141 downregulated) compared to the control. These genes were primarily associated with metabolic, cellular, and stimulus-response processes. The study highlighted that nanoplastic exposure induces oxidative stress, impairs ion and water absorption, and alters various metabolic pathways. Thereby negatively impacting crop growth and yield (You et al., 2026).

## 3 Discussion

Significant progress has been achieved in elucidating the effects of nanoplastics at different stages of the tomato crop (Lakshmikanthan and Chandrasekaran, 2022a; Lakshmikanthan and Chandrasekaran, 2022b; Gao et al., 2023; Cao et al., 2024; Nazari et al., 2025; Sun et al., 2025; You et al., 2026). Most of these studies agree that nanoplastics have adverse effects on the different growth stages of the tomato crop. However, there is also evidence suggesting favorable effects (Cao et al., 2024). Therefore, the knowledge generated to date remains insufficient to fully understand the impacts these nanomaterials can have on this crop. Therefore, we consider it necessary to address the following points to understand the effects of nanoplastics on the tomato crop fully:

### 3.1 Characteristics of nanoplastics

The effects of nanomaterials in different applications are influenced by several of their characteristics, including size, shape, surface chemistry, density, crystalline structure, stability, dispersion, and specific surface area (Shao et al., 2016; Cruz-Luna et al., 2021; Bashir et al., 2023). These characteristics are also critical for understanding the potential effects of nanoplastics on the tomato crop. To date, studies have only partially examined the influence of nanoplastic type, shape, and size on the various stages of tomato crop growth. Therefore, comprehensive studies are needed to systematically evaluate the effects of nanoplastics with varying types, sizes, and shapes on tomato growth, as these characteristics play a central role in determining their biological impacts (Liu et al., 2023). In addition, characteristics—such as surface chemistry, crystalline structure, stability, density, dispersion, and specific surface area— also should be thoroughly assessed to achieve a complete understanding of how nanoplastics affect tomato crop development.

### 3.2 Combinatorial effects

This manuscript focused primarily on the effects of nanoplastic characteristics and concentrations on the different growth stages of tomato cultivation. However, it is essential to analyze the combined toxicity of nanoplastics in interaction with biotic stresses (e.g., pests and plant diseases), abiotic stresses (e.g., drought, salinity, and chilling injury), and other soil pollutants (e.g., microplastics, heavy metals, and residual pesticides), as these factors frequently coexist under agricultural conditions and may exacerbate adverse effects on plant growth and development. Fortunately, some initial studies have partially addressed these combined effects and potential synergistic or antagonistic interactions (Lakshmikanthan and Chandrasekaran, 2022a; Lakshmikanthan and Chandrasekaran, 2022b; Gao et al., 2023; Shi et al., 2023; Cao et al., 2024; Hao et al., 2025; Sun et al., 2025; Zhao et al., 2025). Nevertheless, more comprehensive and systematic studies are still required to fully elucidate the underlying mechanisms and assess the real ecological and agronomic risks associated with these multiple factors.

### 3.3 Methodologies and techniques for quantifying nanoplastics

It is essential to establish standardized methodologies and apply advanced characterization techniques to enable accurate detection and quantification of nanoplastics in tomato crops. These approaches are critical for identifying their presence at different developmental stages and within specific plant tissues. Furthermore, such methodologies and techniques are necessary to elucidate the trajectories and pathways through which nanoplastics are absorbed from the growth medium, translocated within the plant system, and ultimately accumulated in roots, stems, leaves, and fruits. This knowledge will contribute to a more comprehensive understanding of the interactions between nanoplastics and plant cellular components, thereby enabling deeper insight into the molecular and physiological mechanisms underlying nanoplastic toxicity in tomato plants.

### 3.4 Crop factors

The impact of nanoplastics on the tomato crop depends largely on plant-specific factors (Yin et al., 2021). Among the most relevant are the phenological stage, as sensitivity to these materials can vary between germination, vegetative growth, and fruiting. Also, the crop variety is another key factor, as genotypes differ in their physiology, metabolism, and stress response. Similarly, genetic modifications can alter metabolic pathways, defense mechanisms, and photosynthetic efficiency, modulating the plant’s interaction with nanoplastics and their effects on growth, yield, and fruit quality.

In conclusion, significant progress has been achieved in elucidating the effects of nanoplastics at different stages of the tomato crop. However, the knowledge generated to date remains insufficient to fully understand the impacts these nanomaterials can have on this crop. Therefore, it is necessary to address the points described above.
